# Ross, Macdonald, and a Theory for the Dynamics and Control of Mosquito-Transmitted Pathogens

**DOI:** 10.1371/journal.ppat.1002588

**Published:** 2012-04-05

**Authors:** David L. Smith, Katherine E. Battle, Simon I. Hay, Christopher M. Barker, Thomas W. Scott, F. Ellis McKenzie

**Affiliations:** 1 Department of Epidemiology, Johns Hopkins Bloomberg School of Public Health, Baltimore, Maryland, United States of America; 2 Malaria Research Institute, Johns Hopkins Bloomberg School of Public Health, Baltimore, Maryland, United States of America; 3 Fogarty International Center, National Institutes of Health, Bethesda, Maryland, United States of America; 4 Spatial Ecology and Epidemiology Group, Department of Zoology, Oxford University, Oxford, United Kingdom; 5 Center for Vectorborne Diseases, University of California, Davis, California, United States of America; 6 Department of Pathology, Microbiology, and Immunology, School of Veterinary Medicine, University of California, Davis, California, United States of America; 7 Department of Entomology, University of California, Davis, California, United States of America; International Centre for Genetic Engineering and Biotechnology, India

## Abstract

Ronald Ross and George Macdonald are credited with developing a mathematical model of mosquito-borne pathogen transmission. A systematic historical review suggests that several mathematicians and scientists contributed to development of the Ross-Macdonald model over a period of 70 years. Ross developed two different mathematical models, Macdonald a third, and various “Ross-Macdonald” mathematical models exist. Ross-Macdonald models are best defined by a consensus set of assumptions. The mathematical model is just one part of a theory for the dynamics and control of mosquito-transmitted pathogens that also includes epidemiological and entomological concepts and metrics for measuring transmission. All the basic elements of the theory had fallen into place by the end of the Global Malaria Eradication Programme (GMEP, 1955–1969) with the concept of vectorial capacity, methods for measuring key components of transmission by mosquitoes, and a quantitative theory of vector control. The Ross-Macdonald theory has since played a central role in development of research on mosquito-borne pathogen transmission and the development of strategies for mosquito-borne disease prevention.

## Background and Introduction

Mosquitoes transmit the pathogens that cause malaria, filariasis, dengue, yellow fever, West Nile fever, Rift Valley fever, and dozens of other infectious diseases of humans, domestic animals, and wildlife [1]. Physicians and scholars have, throughout history, suspected mosquitoes of transmitting pathogens [2], but the mosquito hypothesis was neither formally tested nor widely accepted until the late 19th century. Patrick Manson, working in China in 1877, was the first to formally demonstrate that mosquitoes transmit a blood-borne pathogen; the filarial worm *Wuchereria bancrofti* was initially isolated from mosquitoes that had fed on his gardener [2], [3]. Charles Laveran observed malaria parasites during 1880 under a light microscope, and several people independently formed the hypothesis that malaria parasites could be transmitted by mosquitoes [4]. Ronald Ross discussed malaria with Manson while in the United Kingdom, but conducted his research while serving in a military post in India, and in 1897 he demonstrated that mosquitoes transmit malaria parasites [4], [5]. Almost immediately thereafter, Ross argued that mosquito population densities could be reduced through larval control and combined with other measures to prevent mosquito-transmitted diseases [6]. He became an important advocate for the public health and economic benefits of control in publications, speeches, and debates [6]–[14]. Meanwhile, in 1900, Walter Reed, Carlos Finlay, and James Carroll showed that mosquitoes transmit yellow fever virus in Cuba, controlled the local *Aedes* mosquito populations, and subsequently stopped transmission [15]–[17]; William Gorgas was sent from Cuba to the Panama Canal to oversee mosquito control to suppress transmission of yellow fever and malaria, leading to the successful completion of the canal [18]. In 1906, Thomas Bancroft showed in Australia that mosquitoes transmit the dengue virus [19]. The successes in controlling mosquitoes and disease in Cuba, Panama, and elsewhere were offset by occasional failures [20], setting the stage for quantitative studies of mosquitoes, pathogen transmission, and control over the decades that would follow.

Of all these important pioneers, Ross casts the longest shadow on mosquito-borne disease because of his contributions to the quantitative theory of malaria and mosquito-borne disease transmission and also to the quantitative foundations of epidemiology (Box 1). In 1904, partly in response to a large, failed larval control trial conducted in Mian-Mir that Ross had debated earlier that year [14], he published a mathematical model describing adult mosquito movement and the spatial scale of larval control required to reduce mosquito populations and eliminate disease from an area [21]. Ross was considering transmission dynamics and control as early as 1902, but did not publish his first malaria transmission model until 1908 [22]. He published a second malaria transmission model in 1911 in an addendum to his book, *The Prevention of Malaria*, and described it in *Nature* [23], [24]. Ross's last original contribution to modeling malaria, in 1921, discussed the value of repeated drug treatment to “cure” malaria infections [25].

**Box 1. Ross's *A Priori* Pathometry and Mathematical Epidemiology**

Ross's malaria models alone would have earned him a place in history, but he was also instrumental in establishing the intellectual foundations for the study of disease dynamics. Ross was not the first to model an infectious disease; indeed, several early papers had already established the foundations of epidemiology. John Snow had published the classical study of cholera in 1855 [99] and several quantitative, but mainly statistical, studies in epidemiology followed Snow at the end if the 19th century. Ross's mathematical ideas also had precursors. Daniel Bernoulli developed a dynamic model of smallpox transmission and control in 1760 [100], a remarkable study of disease transmission dynamics had been published by En'ko in Russian in 1889 [34], [101], and Hamer published a measles transmission model in 1906 [102]. Ross's aspirations were not just to understand malaria, but also to establish a new branch of science. In 1908, when he published his first dynamic malaria model, Ross coined the phrase “*a priori* pathometry” to describe the scientific activity of modeling transmission dynamics, and in 1911, he presented a new set of equations as part of a general framework [23], [24]. Ross's second malaria model was a special case of his new, general theory: he called malaria a “metaxenous” disease. In 1915, he solved the general equations, and discussed his work in relation to Brownlee's [103], who was developing a complementary set of methods for studying epidemics [33]. Both men used the terms *a priori* and *a posteriori* to describe two different approaches to studying epidemics, though they switched the meanings [31]. In 1916, Ross published the first of a three-part series laying out the expanded theory of *a priori* pathometry [104]. Ross described the *a priori* method, “we assume a knowledge of the causes, construct our differential equations on that supposition, follow up the logical consequences, and finally test the calculated results by comparing them with the observed statistics,” and the *a posteriori* method, “we commence with observed statistics, endeavour to fit analytical laws to them, and so work backwards to the underlying cause (as done in much statistical work of the day).” Ross argued that epidemics were, *per se*, a phenomenon worthy of study. Ross believed that the study of epidemics was intrinsically quantitative and that epidemics were extremely complicated, so understanding them would require a combination of mathematical modeling based on *a priori* notions of cause and examination of patterns in data through statistical investigation [104]. The last two parts were co-authored by Hilda Hudson and published in 1917 [104]–[107]. In 1927, Kermack and McKendrick published the first [108] of their seminal papers [109]–[112]; McKendrick had been with Ross in Sierra Leone, and his work acknowledges the contributions of Ross and Hudson. Ross called the field “*a priori* pathometry”, or “constructive epidemiology” [113], but it is now more widely known as mathematical epidemiology.

Ross recognized that a complete quantitative theory needed methods for measuring transmission, but while he made great conceptual advances and helped develop new parasitological methods, he had not developed useful metrics for measuring the important components of transmission by mosquitoes. Ross's ideas motivated a generation of medical entomologists, and starting in 1950, there was a great leap forward due to the theoretical work of George Macdonald and the empirical work of some of his close associates. With these contributions, the theory for transmission dynamics and control had all of its elements and the links between the models and the metrics had been made. The “Ross-Macdonald” model became firmly established as a basis for a broader theory of mosquito-borne disease transmission and control. The model has played the classical role of a scientific theory; it is a deliberately simplified set of concepts that serves as a basis for studying mosquito-borne pathogen transmission. Like other theories, it has formed the starting point for a dialogue about methods, for defining what should be emphasized and measured, and for building new models of mosquito-borne disease transmission. The Ross-Macdonald models influence continues to the present day.

The Ross-Macdonald theory and its development is often misunderstood in its historical context, cited incorrectly, or simply forgotten. This article describes the historical development of basic models and concepts for mosquito-transmitted pathogens starting with Ross and following it through Macdonald's seminal contributions, the maturation of the theory around 1964, and a few key subsequent papers. A comprehensive bibliography of modeling papers is annotated and published as an online supplement (Text S1) and notation conventions are described in Box 2 and aligned in Table 1. This paper and the bibliography have benefitted from histories or commentaries written by Lotka [26], Bailey [27], [28], Bruce-Chwatt [18], [29], [30], Fine [31]–[33], Service [2], Dietz [34], [35], Molineaux [36], Koella [37], and McKenzie [38]. The field of modeling mosquito-borne pathogen transmission since the late 1960s has developed too rapidly and extensively to be described simply. A comprehensive review and systematic analysis of more recent developments is being prepared as a future companion to this paper.

**Box 2. Notation**

Several quantities are commonly defined as part of the Ross-Macdonald model; the population density of humans, *H*; the population density of mosquitoes, *M*; the number of infected humans, *X*; the number of infected, but not yet infectious mosquitoes, *Y*; the number of infectious mosquitoes, *Z*; the human blood feeding rate, the proportion of mosquitoes that feed on humans each day, *a*; mosquito survival as either the probability of surviving one day, *p*, or the instantaneous death rate, *g* (*p = e^−g^* or *g* = −ln *p*); the pathogens' vertebrate latent period, often called the “intrinsic incubation period”, the number of days from infection to infectiousness in the human, *u*; the pathogen's mosquito latent period, often called the “extrinsic incubation period”, the number of days from infection to infectiousness in the mosquito, *v*; the daily rate each human recovers from infection, *r*; the proportion of infected humans that are infectious, or alternatively, the probability a mosquito becomes infected after biting an infected human, *c*; and the proportion of bites by infectious mosquitoes that infect a human, *b*. It is also sometimes useful to consider the human blood feeding rate as the product of a blood feeding rate, *f*, and the fraction of blood meals on humans, or more generally, the pathogen's host, *Q (a = fQ)*.

Important and measurable quantities can be recognized in models including: the prevalence of malaria, malaria rate, or parasite rate (*x = X/H*); the fraction of infected but not infectious (*y = Y/M*) or infectious mosquitoes (*z = Z/M*); the ratio of mosquitoes to humans (*m = M/H*); the number of bites by vectors per human per day, called the human biting rate (HBR, *ma*), the number of infectious bites per human per day, called the entomological inoculation rate (EIR, *maz* or *E* in equations); the force of infection or “happenings” rate for human infections (*h = mabz*); the average lifespan of a mosquito (*1/g*), the number of human bites per mosquito over its lifespan, called the stability index (SI, *a/g* or *S* in equations); the probability an infected mosquito survives to become infections (*P = e^−gv^*); the average number of days a person remains infected (*1/r*), the net infectiousness of humans to mosquitoes, the probability a mosquito becomes infected after feeding on a human (*κ = cx*), the force of infection or “happenings” rate for mosquito infections (*aκ*). Formulas are given in the main text for the vectorial capacity (*V*) or daily reproductive number and basic reproductive number (*R_0_*) and the critical density of mosquitoes required for sustaining transmission (*m′*).

Each version of the Ross-Macdonald model has used a subset of these parameters, but each one has also utilized a different notation. Several of these models have been described in the boxes using the common notation defined above. The notation originally used in the models has been aligned with this notation in Table 1.

**Table 1:** Alignment of notation.

	Box	Parameter Names																			
Common Notation	2	*M*	*H*	*m*	*X*	*x*	*Z*	*z*	*y*	*a*	*g*	*r*	*c*	*b*	*u*	*v*	*h*	*κ*	*aκ*	*P*	*R_0_*
Ross (1^st^) [22]	3	*a*	*p*			*m*				*f = bp = ¼*		*r*	*i = ¼*							*s = ⅓*	
Waite [41]	3	*a*	*p*		*m*					*f = bp = ¼*		*r*	*i = ¼*							*s = ⅓*	
Lotka [42]	3	*a*	*p*			*m*				*β*		*r*	*i*							*S*	
Ross (2^nd^) [23]	4	*p*	*p′*		*z*		*z′*					*q*					*k′z′*		*kz*		
Sharpe & Lotka [46]	5	*p*	*p′*		*z*		*z′*					*q*			*u*	*v*	*k′z′*		*kz*		
Macdonald [55], [67]	6			*m*		*x*				*a*	*−ln p*	*r*		*b*		*n*		*x*		*p^n^*	*Z_0_*
Aron & May (1^st^) [91]	7	*M*	*N*			*x*		*z*		*a*	*μ*	*r*		*b*				*x*			
Smith & McKenzie [93]	7			*m*		*x*		*z*		*a*	*g*	*r*	*c*	*b*		*n*		*cx*		*e^−gn^*	
Aron & May (2^nd^) [91]	7	*M*	*N*			*x*		*z*	*y*	*a*	*μ*	*r*		*b*		*τ*		*x*		*e^−gτ^*	
Anderson & May [92]	7	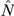	*N*			*y*		*ŷ*		*a*	*μ*	*γ*	*c*	*b*							

Each version of the Ross-Macdonald model used different parameter names for the same or very similar quantities. This table aligns all of those names. The common notation is defined in Box 2. Differences in the parameter interpretations described in the separate boxes.

## The Birth of a Theory: 1899–1949

For Ross, quantitative thinking came naturally. Mathematical models were a way to codify, refine, and communicate the quantitative logic of biological phenomena, especially mosquito-borne pathogen transmission, in a form that was rigorous and testable. In his correspondence with Manson in 1897, before successfully demonstrating that mosquitoes transmit malaria, Ross was already reasoning quantitatively about his own fever [39]:

An incubation period of two or three days…simply implies to mathematical demonstration an access or ingress of many millions of parasites at the moment of infection. Now whence does this invading host come? Are they at the moment of infection (a) multiplying free in nature or (b) parasitic in some other animal. (pp. 163–164)

Two years later, Ross wrote about the extermination of mosquitoes [6]:

…in order to eliminate malaria wholly or partly from a given locality, it is necessary only to exterminate the various species of insects which carry the infection. It remains only to consider whether such a measure is practical. Theoretically, the extermination of mosquitos is a very simple matter.

Some of his preliminary thoughts about modeling were also apparent in 1902, when he speculated about the mathematical laws of transmission [13]:

It may now be asked, what percentage of diminution in mosquito-borne disease may be expected to follow a given percentage reduction in the number of mosquitoes? I regret that I cannot as yet give any actual statistics on the point, but we may perhaps attempt an estimate on a priori grounds…. If we reduce the number of mosquitoes in the locality by one-half, the mosquito bites will be reduced by one-half; and consequently, only half as many people will now become infected as was formerly the case. But, since the mosquitoes themselves are infected by biting previously infected persons, the percentage of infected mosquitoes, among the insects which remain, will also be reduced in its turn, because the insects will now find fewer infected persons to bite. Hence, ultimately, the number of mosquitoes will be reduced by much more than one-half. In fact, we may perhaps assume that the number of infected persons will be reduced to one-quarter – that is, in the duplicate ratio of the squared percentage of the reduction of the mosquitoes. (p. 56)

The reasoning is similar to the transmission models he formulated 6 years later, and shows he was already thinking about transmission in quantitative terms. In his critique of the experiment at Mian-Mir, he wrote [14]:

…the broad principles which govern the prophylaxis of malaria… though self-evident enough, require a more or less mathematical treatment for their formal demonstration… Experiment is required, not in support of the general principle, but only in order to obtain certain unknown constants.

At the time, the methods did not yet exist to describe malaria transmission mathematically, to measure the relevant constants, or to know what those constants were.

Ross's first model is, in many ways, an extended critique of the experiment at Mian-Mir, but it did not directly address the question of transmission. The model itself describes random movement of adult mosquitoes in and out of concentric zones surrounding the center of an area that had been completely depleted of aquatic habitat. Ross's analysis of the model suggested that adult mosquito densities would decline outside the edge of a control zone as mosquitoes wandered into the non-control area. The process would create a sigmoidal gradient in mosquito density, and if the control zone was large enough, an area in the middle would be mosquito-free [21]. Ross concluded that larval control could work if it could deplete larval mosquitoes in a large enough area; but no conclusions about the validity of larval control, *per se*, could be reached if it had not been done intensively enough, for long enough, at a large enough scale.

After Ross visited Mauritius in 1907 to advise on the control of malaria, he formulated and described a model of mosquito-borne disease transmission in 1908 in his *Report on the Prevention of Malaria in Mauritius* (pp. 30–37 in [22]), and he expanded on these ideas in the first edition of *The Prevention of Malaria* [40]. The model was an *a priori* description of the number of infections in humans based on his quantitative reasoning about the number of mosquitoes and their infection dynamics. It can be formulated as a difference equation (Box 3). At Ross's invitation, Waite analyzed the model and wrote a clear description of the model assumptions and limitations [41]. The model was concisely presented and analyzed again by Lotka [42]. Ross's main conclusions from the models were that there is a causal relationship between the ratio of mosquitoes to humans and the number of infected humans, and that it was not necessary to kill every mosquito to end transmission. The models demonstrated that there was a critical mosquito density, 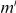, such that greater densities would sustain transmission while lesser ones would not. Ross's formula (making some liberal allowances in the interpretation of parameters) is equivalent to the following:
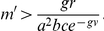
Ross was unsatisfied with some minor numerical discrepancies between his results and Waite's. These discrepancies arose because they had picked different time steps for simulation. Ross then set about to reformulate a general model that would not depend on any particular time step. He formulated the model using a system of coupled differential equations in continuous time (Box 4); though mathematically different, the second model was the limiting case of Ross's first model with an infinitesimally small time step. At the same time, he wanted to develop a more expansive theory. Ross's second malaria transmission model was published as an addendum to the second edition of *The Prevention of Malaria* in 1911 [23] and in *Nature* [24].

**Box 3. The Ross-Waite-Lotka Model**

Ross's first dynamic model of malaria [22] was further developed by Waite [41] and Lotka [42]. Lotka wrote the model more elegantly as a simple difference equation: 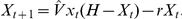
Ross formulated a quantity, here called 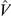, that is very similar to vectorial capacity. The derivation is very similar, but there are some differences. Ross's time step was one month, and his formula considered at most two bites per mosquito each month, one that infected it and one that transmitted the parasites. Thus, in the alignment of notation (Box 2), the interpretation of Ross's *f* (or equivalently *bp*) is not identical to the human blood feeding rate, *a*. Waite's time step was the interval between bites, but he retained the interpretation of *f*.

**Box 4. The Ross-Lotka Model**

The second dynamic model of malaria was published by Ross twice in 1911, first as an addendum to the second edition of *The Prevention of Malaria* [23], and then in *Nature* [24]. One year later, Lotka proposed a closed-form solution [43], and in 1923, Lotka thoroughly analyzed it [42], [44], [45], [114]. The model formulation was more focused on mathematical details, and not on the entomological ones. The parameters here have been supplied from alignment (Table 1):
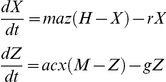
It must be noted that Ross also considered births and deaths in both the human and vector populations, but he set these equal to each other so the populations would be in their steady state for analysis.

Lotka solved Ross's second model in 1912 [43], and in 1923, Lotka published a five-part analysis of Ross's malaria models. The first analyzed Ross's second model [44] and the second showed how Ross's first two models were related [42]. Lotka's third paper in the series included a comprehensive numerical analysis, a diagram of the phase-plane, and a photograph of a clay model that interprets the phase plane as a topographic surface [45]. In the fourth, Sharpe and Lotka extended Ross's second model (Box 5) and considered the pathogen's latent period in the mosquito, commonly called the extrinsic incubation period, and the pathogen's latent period in the human or other vertebrate host, or the intrinsic incubation period [46]. Altogether, Lotka's five-part analysis and extension of Ross's original models represented a landmark achievement in the mathematical analysis of mosquito-borne disease models.

**Box 5. The Sharpe-Lotka Model**

Sharpe and Lotka [46] extended Ross's model to consider the latent period in both humans and mosquitoes:
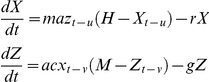
The analysis is focused on mathematical details, not biological ones, and so the model neglects mosquito mortality during the latent period, and so they conclude that the delay has no effect on the equilibrium.

Ross also used malaria models to reason through several different kinds of problems. He frequently discussed control, as he had done in 1902, but he did not formally model it. He argued that multiple modes of control would often be necessary, including larval control, bednets, improved housing, and “segregation of the races.” He also argued, informally, that these interventions were inexpensive relative to the enormous health benefits of control, foreshadowing later arguments about cost-effectiveness. Ross understood that operational concerns were important [13], [20], [22], [40]. The real question was whether control could be done efficiently enough. Ross understood the complex, non-linear nature of epidemics, their interplay with immunity, and the problems with observational data, and he used models to explain how counter-intuitive patterns (i.e., causation without an apparent correlation) could arise [24].

Ross developed the models to be part of a general quantitative theory for malaria epidemics [23], [24] (Box 1). Part of this theory would require measuring transmission, and Ross either advanced malariometric methods through his own work as well as his influence on later scientists. Dempster had introduced the use of the spleen rate as an index of malaria transmission in 1847 [47], and Laveran had already identified the parasites. At the time, the spleen rate was more widely used than microscopy, even though a diagnosis through microscopy was more specific. Ross improved microscopy further by developing the “thick film” to increase the sensitivity of parasite detection by light microscopy [48], [49]. With Thomson, Ross used the thick film to count parasites over the course of an infection and find an association with clinical symptoms [50]. Over time, the advantages of the thick film were recognized [51], and it became more widely used as a diagnostic tool. Ross called the prevalence of parasites by light microscopy the “malaria rate,” but later the “parasite rate” came into common usage. This diagnostic is still utilized routinely across the globe [52].

Ross's notions had, in some sense, been at the heart of early efforts to measure malaria transmission entomologically. The expedition to Sierra Leone in 1899 had focused much of its attention on the vector populations, but Ross acknowledged that the expedition ended without developing metrics for measuring key components of a mosquito's role in pathogen transmission [14], [21]. His first model of transmission describes at most one pair of bites for each mosquito, but it does not quantify important details such as mosquito lifespan and blood feeding behavior, and he did not update the entomological components in the second model. The lack of an entomological measurement of transmission was a major shortcoming of Ross's theory.

By 1930, field studies had advanced substantially. Davey and Gordon, who were aware of Ross's theory and motivated by his ideas, informally compared multiple kinds of epidemiological and entomological data to test Ross's notions, and especially to identify which vectors were most important for transmission [53]. They measured the “infective mosquito density,” an early name for measures of the number of infectious mosquito bites per person per day, later called the entomological inoculation rate (EIR) [54]. They also plotted the parasite rate stratified by age. The difference between the counted infectious mosquitoes and the number of infections observed in humans was already so stark that the authors could note from visual inspection that there was a good qualitative but poor quantitative correspondence. Other entomologists also tested Ross's theory of a critical density of mosquitoes and advanced the field methods [55].

Ross also recognized the value of measuring malaria transmission by looking at the incidence of malaria in people who were new to the area [13]. He later developed a quantitative theory of “happenings,” which was his name for the “force of infection,” or the hazard rate for infection [23], [24]. He had proposed that there was a connection between mosquito densities and the number of infections, but this idea was disputed by the lack of a crude association between mosquito densities and malaria fevers. Ross used models to illustrate several factors that could explain the gaps [24], and as early as 1902, he had recognized the value of counting infections in previously unexposed populations [13]. The theory of happenings was first outlined in 1911, but in 1915 he “solved” the equations describing the proportion infected in a cohort of a given age. He was not the first: the equations were an alternative form of the logistic curve and they had been applied to epidemic data and solved earlier by Bernoulli [34].

A few years later, Muench developed these ideas further [56]–[58] and presented a general discussion of equations and methods for the statistical analysis of the kinds of age-prevalence curves being collected by Davey and Gordon and others studying malaria, age-seroprevalence data being collected by Soper for yellow fever, and other diseases [56], [57]. These were later codified with the analysis of multiple datasets in the 1959 book *Catalytic Models in Epidemiology* [58].

## The Ross-Macdonald Theory Matures, 1950–1969

Ross had focused on malaria control, but at the time, there were no alternatives for lasting control of adult mosquito populations. This changed with the discovery of the insecticidal properties of DDT in 1939, when it became possible to kill adult mosquitoes for several months by spraying the insecticide once on the interior walls of houses. After World War II, DDT was used in large-scale typhus and malaria control programs. The World Health Organization (WHO) was founded during 1948, and Fred Soper and others began to argue for the global eradication of malaria [59], [60] and for the eradication of *Aedes aegypti* from the Western hemisphere. Relatively soon thereafter, the Global Malaria Eradication Programme (GMEP, 1955–1969) was formally launched by a vote at the eighth World Health Congress. Since Ross had first published his models, there had been several decades of epidemiological and entomological field studies, including the ones by Davey and Gordon and the statistical methodological advances by Muench. These developments set the stage to extend Ross's earlier work.

George Macdonald led the effort. Macdonald had followed, quite literally, in Ross's footsteps. He conducted a field study of malaria in Sierra Leone where Ross had gone in 1899, and from 1947, he was Director of the Ross Institute. In 1950, he turned his attention to the mathematical theory of malaria transmission.

The Ross-Macdonald theory of mosquito-borne pathogen transmission, so-named, often gives the impression that Macdonald dramatically enhanced Ross's models mathematically. Macdonald did, in fact, innovate mathematically on Ross's model by introducing superinfection, reinfection of those who are already infected so that they carry multiple parasite types [61] (Box 6), but his most important contributions were to develop the entomological theory and the quantitative theory of control that Ross had been attempting a half-century earlier. Macdonald also used the models to quantitatively synthesize a half-century of malaria epidemiology, which made it possible for the two-way flow of ideas to occur between theory and entomological and epidemiological data.

**Box 6. Macdonald, Irwin, Dietz, and Superinfection**

Macdonald's complete model was presented in a series of papers [55], [67], [71], and except for the original paper on superinfection, usually relegated to brief summaries in the appendices of his papers. The model he uses is essentially the following:
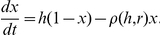
The “happenings” rate is defined by the formula:
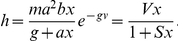
Macdonald (with Irwin) first defined a function describing the recovery rate under superinfection. The mathematical model is perfectly valid, but it was not consistent with the process they described of individual infections being acquired and clearing independently [32]. This process was later described correctly by Dietz in the Garki model [97]. Here it is paired with the simpler formulation to become the Mcdonald-Dietz model.
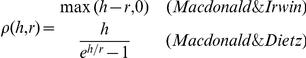
Macdonald simulated epidemics [78]. In so doing, he used equations similar to Ross's first model (see Box 3):
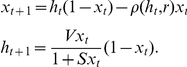

Ross had assumed that infections were simple—the infection must be cleared before a new infection can occur—but early malaria research made it clear that superinfection was common. Ross even discussed multiple infections in 1911 [23]. McKendrick had studied the issue in some detail and formulated a theory for the distribution of the number of events occurring in a fixed interval of time as well as for changes in the multiplicity of an event or infection [62]–[65]. By 1947, Walton had used a Poisson distribution to model the multiplicity of infection with malaria, i.e., the number of distinct parasite types carried simultaneously in the blood [66]. Macdonald's first mathematical publication extended Ross's models to consider dynamic changes in clearance rates under superinfection [67]. There was, however, a discrepancy between Macdonald's mathematical formulas and his written descriptions of them [32]. Macdonald's description of the model with distinct parasite broods clearing and being acquired independently agreed with earlier formulations, including Walton's, but the mathematics described a different process. As recounted by Fine, the discrepancy was due to a miscommunication with Irwin, who had helped derive the model [32].

In a paper that was published as a companion to the superinfection model, Macdonald used the catalytic models of Ross and Muench to analyze the data collected by Davey and Gordon and others [68]. Even though his methods were poorly documented and the technical merits can't be assessed in modern terms, he accomplished several “firsts,” including a first estimate of the “recovery” rate from malaria infection before Eyle's analysis of malaria-therapy data [69], and a first estimate of the force of infection using age-stratified parasite rate data for malaria.

In 1952, Macdonald turned his attention to the entomological theory of transmission. He assembled a half-century of entomological field data describing mosquito survival, blood feeding, and the relationship between temperature and the extrinsic incubation period for *Plasmodium falciparum* and *P. vivax* [55]. A critical insight was the quantitative importance of mosquito longevity, which Macdonald first published in 1952 and again in 1956 as a theoretical justification for using DDT for malaria eradication [55], [70]. In 1952, Macdonald also expanded on Ross's notions of mosquito density and biting, and he developed an entomological theory of malaria transmission based on the mosquito feeding cycle and demography (see Box 2). In a follow-up paper, Macdonald borrowed Lotka's demographic concept of a basic reproduction ratio for malaria [71]. Later, he called the quantity *Z_0_* [72], but it is now more commonly called *R_0_* and the name has become a standard throughout mathematical epidemiology. *R_0_* describes the expected number of hosts that would be infected by a single infected host in a completely susceptible population:
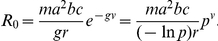
Macdonald's attention to decades of epidemiological studies facilitated development of methods to measure transmission entomologically: one year later, Draper and Davidson published the first estimate of *R_0_* [73]. Draper, Davidson, and Gilles also combined the ideas from Macdonald's models and mosquito natural history and used mosquito parity, the proportion of mosquitoes that had laid eggs at least once, to estimate mosquito longevity [73]–[77]. These landmark papers paved the way for the expansion of an entomological theory that would soon come. Over the next few years, Macdonald wrote papers discussing *R_0_* in relation to both endemic and epidemic malaria [71], [72], [78] (see Text S1).

As enthusiasm for the GMEP built, Macdonald's work was laying a mathematical foundation for eradication that emphasized measuring transmission and control [79]. While Ross's theory had focused on larval control, Macdonald's problem was transmission by and the attack on adult mosquitoes with DDT or other contact pesticides. The first indoor residual spraying programs utilizing DDT had been remarkably successful and helped make the case for eradication [59]; the quantitative basis for that success was explained through a sensitivity analysis on adult mosquito longevity [70]. Macdonald is often wrongly credited with being the first to mathematically incorporate the pathogen latent period in the mosquitoes—as mentioned, credit for this goes to Sharpe and Lotka [46]. Macdonald developed a useful formula for mosquito mortality during sporogony, but he also gives credit to Armitage for advancing the mathematical ideas about the delay [80]. Even so, Macdonald recognized the epidemiological importance of the latent period in the mosquito and mosquito longevity when seen in light of adult mosquito control [55], [70]. The sensitivity analysis had showed that the reductions would affect transmission non-linearly. With the beginnings of a theory of control in place, Macdonald was able to explain the rationale for measuring transmission for eradication, as part of the GMEP [70], [79]. The ideas were collected and synthesized in his book, *The Epidemiology and Control of Malaria* [81].

WHO entomologists led by Garrett-Jones further developed methods for measuring transmission entomologically. They gave the name *vectorial capacity*, or alternatively the “daily reproduction rate”, to the purely entomological concepts of *R_0_*, and it was defined as the expected number of infective mosquito bites that would eventually arise from all the mosquitoes that would bite a single fully infectious person on a single day:
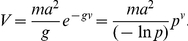
Vectorial capacity describes the potential intensity of transmission by mosquitoes. More importantly, they codified a set of methods for measuring feeding rates and the human blood index [82]–[84]. For decades, entomologists had been counting infectious mosquitoes in the proximity of humans and using it as a measure of risk under various names [53]. In 1980, the estimated number of infectious bites per person per day was renamed the EIR [54]. The quantity was closely related to both vectorial capacity and “happenings”, or force of infection (Box 2). These methods have since expanded and are now a standard part of mosquito field sampling methodology (see chapter 13 in [85]).

**Figure 1:**
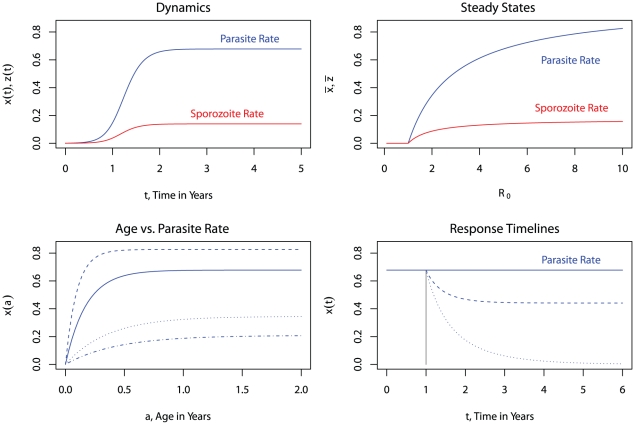
The Ross-Macdonald theory of transmission dynamics. (Top left) In a hypothetical location, for a fixed value of *R_0_* (plotted here for *R_0_* = 5), the model describes changes in the proportion of infected humans or infectious mosquitoes during an epidemic. (Top right) Alternatively, the models predict the endemic parasite rate or sporozoite rate as a function of *R_0_*. Malaria is not endemic if *R_0_*<1, or after control, if *R_C_*<1, or equivalently, if mosquito density is below a critical threshold. (Bottom left) The model also describes changes in the parasite rate with respect to age (e.g., in a cross-sectional study) in infants or others who were previously unexposed to malaria. (Bottom right) Finally, the models also predict the response timelines and endpoints following the implementation of control (grey).

**Figure 2:**
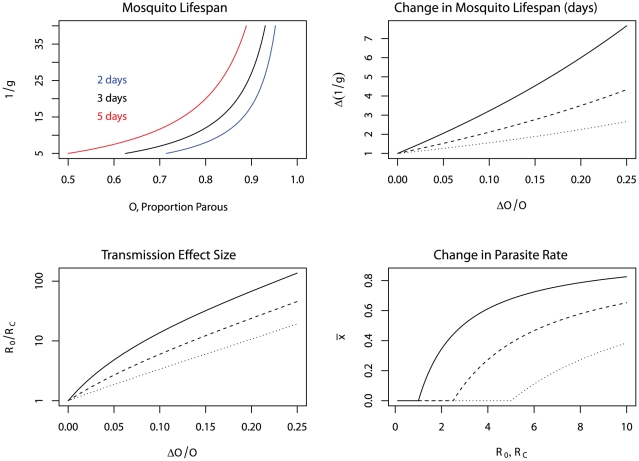
The Ross-Macdonald theory of control. (Top left) A relationship exists between the length of a mosquito feeding cycle (2, 3, or 5 days in blue, black, or red), the proportion of parous mosquitoes (denoted *O*), and the mosquito lifespan (denoted *1/g*). (Top right) This relationship can be used to measure predicted changes in the mosquito lifespan (*Δg^−1^*) through estimated proportional changes in the proportion parous, which are invariant to the mosquito blood feeding rate (*ΔO/O*). (Bottom left) These changes can be translated into an effect size on transmission, a proportional change in reproductive numbers (*R_0_/R_C_*). (Bottom right) Finally, these can be translated into changes in the endemic parasite rate for a given effect size: *R_C_* = *R_0_*/2.5 (dashed) or *R_0_*/5 (dotted).

Collectively, these ideas paved the way for measuring transmission and control by vectors that made an explicit connection between the proportion of parous mosquitoes before and after control, the proportional reductions in vectorial capacity or *R_0_*, and a comparison between model predictions for the likelihood of elimination or changes in endemicity and the actual outcomes [84]. The predictions of the theory were put to the test in 1969 in a study that measured vectorial capacity, estimated the control effect sizes of DDT, and examined the predicted versus actual changes in endemicity [86]. This was, finally, a synthesis of the ideas sought by Ross and partly described by Macdonald (Figures 1 and 2).

The theory was applied extensively during the GMEP. Macdonald had played a role in debates about malaria control in Africa during a historically important conference in Kampala in 1950, siding with Soper and arguing for scaling up control in Africa [59]. As the GMEP established its programmatic form and timelines, Macdonald's analysis and insights helped give those ideas a quantitative rigor through his advisory role on definitive technical documents. He served as rapporteur for the *Sixth Report of the Expert Committee on Malaria* published by the WHO [87], the document that lays out the four phases of a malaria elimination program, including 3- to 5-year endemicity response timelines for the attack phase. A decade after the GMEP started, Macdonald refined the theoretical basis for endemicity response timelines and measures of successful interruption of transmission [88].

Macdonald had described the mathematical basis for changes in endemicity, the relationship between endemicity and *R_0_* [71], and the response timelines for the interruption of transmission following control [88], but most of his work was focused on a theory of elimination following an overwhelming reduction in transmission. In 1964, another WHO mathematical epidemiologist, named Moskovskij (aka Moshkovsky), explored changes in endemicity after the implementation of control at levels too low to interrupt transmission [89] (Figure 1). Moskovskij described his theory in terms of “communicability,” something like the EIR or the force of infection or vectorial capacity, and the “exhaustibility”, the recovery rate or the inverse of the duration of an infection. The product of these two was, in Moskovskij's description, equal to *R_0_*. Moskovskij then related changes in transmission intensity achieved through malaria control to changes in malaria endemicity.

Macdonald's final theoretical contribution, published after his death, was a stochastic model of malaria transmission, including the first simulations of a mosquito-borne pathogen ever conducted on a computer [90].

## The Ross-Macdonald Theory, 1970 to the Present

Having described the history of an idea developed by Ross, Macdonald, and others, it would be useful to present “The” Ross-Macdonald Model, but no canonical mathematical formulation exists. There are, instead, several different models and types of modeling styles that are commonly called “Ross-Macdonald” models and several historical precedents including the Ross-Waite-Lotka model (Box 3), the Ross-Lotka model (Box 4), the Sharpe-Lotka model (Box 5), and the Macdonald-Irwin and Macdonald-Dietz models (Box 6). Several alternative versions have been published since Macdonald [91]–[93] (Box 7). In 1974, Fine published critical reviews of the models by Ross [31] and Macdonald [32]. In 1957, Bailey republished Ross's second model in *The Mathematical Theory of Epidemics* [27], and in 1982, Bailey wrote a comprehensive review of the Ross-Macdonald model in *The Biomathematics of Malaria* [28] with separate chapters presenting the work by Ross and Macdonald, and another describing a general theory. Ross's second model was a very simple compartment model, but Bailey expanded it, presenting a general theory of mosquito-borne disease transmission as an SIR-SI model. In 1991, Koella described several models for malaria [37], and in 1992, Newton and Reiter published an SEIR-SEI model for dengue [94], and various versions of these compartment models are increasingly being used and called “Ross-Macdonald” style models.

**Box 7. Ross-Macdonald Style Models**

Several models have been published as a Ross-Macdonald model. In 1982, Aron and May first wrote it in the following way [91]:
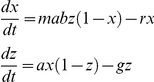
This model considers infected but not infectious mosquitoes, so it ignores the delay for pathogen latency in mosquitoes. There are several ways to consider the delay or its effects. Smith and McKenzie wrote down a simple model with two equations that does incorporate mosquito mortality during the latent period but that ignores the delay [93]:
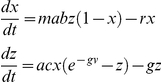
Aron and May also formed a second model, a delay differential equation that is, perhaps, the best simple implementation of the Ross-Macdonald model [91]:
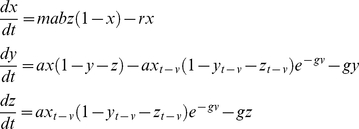
Later, Anderson and May wrote down the following version of the Ross-Macdonald model [92]:
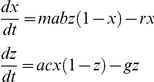

Without a canonical formulation, the “Ross-Macdonald model” is more usefully described as a set of models all based on a consensus set of simplifying assumptions, and in its development, these ideas are inextricably linked to a set of methods for measuring transmission epidemiologically and entomologically. A Ross-Macdonald model is based on a simplified process-based quantitative description of the pathogen life cycle in four steps: (1) the pathogen is passed from an infected mosquito to a vertebrate host during blood feeding; (2) it infects and then multiplies in the vertebrate host, reaching sufficiently high densities in peripheral blood to infect a new mosquito; (3) a susceptible mosquito imbibes the pathogen from the infected vertebrate host during blood feeding; and (4) the pathogen develops in the mosquito to a point that it is in the salivary glands or mouth parts and ready to be transmitted during a subsequent bite on a susceptible vertebrate host. Infection dynamics in the mosquito are based on a simplified description of the mosquito cycle of blood feeding and egg-laying. The models differ in the species of mathematical model and in the way they implement latency in the mosquito, but there is a consensus set of simplifying assumptions about the transmission dynamics: mosquito bites are distributed randomly and evenly among vertebrate host populations, populations are closed to birth or migration (except Ross's second model), there are many more humans than infectious bites, there is one vertebrate host (usually humans), human infections are simple and clear at a constant per-capita rate (except Macdonald's model), hosts become susceptible to infection after recovery (until chapter 6 in Bailey [28]), the ratio of mosquitoes to humans is constant (until Aron and May [91]), mosquito mortality is independent of age so that the mosquito lifespan is exponentially distributed, the pathogen latent period in mosquitoes is constant, there is only one mosquito vector species, and a constant fraction of mosquitoes blood feed on the pathogen's host.

The Ross-Macdonald theory of control is based around the notions of *R_0_* and vectorial capacity, which vary over space and time, depending on differences in adult mosquito abundance, longevity, biting rates, human blood-feeding habits, and the pathogen's latent period in the mosquito [82]. Vectorial capacity is expensive and time consuming to measure, but it is closely related to the EIR. Under the consensus assumptions and notation, it is possible to rewrite one equation (from Smith and McKenzie [93], Box 7) describing the change in EIR (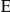
in equations; for other notations see Box 2) with respect to vectorial capacity:
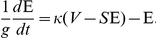
Vectorial capacity and EIR change on timescales determined by the mosquito lifespan, and they are closely related concepts that provide complementary measures of vector transmission and a basis for evaluating vector control. The difference between the number of infectious bites received by the typical host (i.e., EIR) and infectious bites that could potentially arise from fully infectious hosts (i.e., vectorial capacity) is due mainly to the low actual infectiousness of the reservoir (i.e., 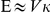).

The logic of control in the Ross-Macdonald model focuses on *R_0_*, which describes maximum transmission potential. If the pathogen is present and *R_0_*>1, there will be an epidemic in the absence of control, and if conditions remain constant, the fraction of infected humans and infectious mosquitoes will reach a steady state (Figure 1). Under some form of control, maximum potential transmission is described by a lower effective reproductive number, *R_C_*, which is analogous to *R_0_* in every way except that it is subject to the limits of control. If *R_C_*>1, a pathogen will tend to remain endemic, but if *R_C_*<1, then infections fail to replace themselves and a pathogen will be eliminated on timelines that depend, in large part, on the magnitude of *R_C_* [88]. *R_0_* and *R_C_* thus describe a framework for setting intervention control targets for elimination: *R_0_* describes the total proportional reductions in transmission that must be achieved and maintained through various modes of control to interrupt endemic malaria transmission; the total “effect size” already achieved with a set of interventions is given by *R_0_/R_C_*; and *R_C_*>1 describes a shortfall that must be made up with increased coverage, new interventions, or new tools. Immunity can also suppress transmission, so potential transmission where pathogens are endemic reflects the combined effects of control and immunity. Combining the analysis of Ross, Macdonald, Moskovskij, and medical entomologists, there are well-defined quantitative relationships between *R_0_* and vectorial capacity, the EIR, the effect sizes required to interrupt transmission or reduce the parasite rate, the incidence of malaria in those who are previously unexposed, and the rise in the parasite rate with age (Figure 1).

A reformulation of *R_0_* clarifies the effects of control in the Ross-Macdonald theory (Box 8). First, the effects of different modes of control typically affect different terms [37], with the result that effect sizes achieved through different means of integrated control are multiplicative. In other words, a 10-fold (i.e., 90%) reduction in transmission achieved through adult vector control combined with a 5-fold reduction in transmission achieved with a vaccine (i.e., 80%) would have a total effect size of 50 (i.e., 98%), and this would interrupt transmission wherever *R_0_* was less than 50.

**Box 8. Integrated Control**

For the purpose of describing control effect sizes of different interventions alone or in combination, it is more useful to write *R_0_* in a slightly different, but equivalent way. Let 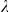 denote the number of adult mosquitoes that are born each day, divided by the population density of humans. Under the consensus assumptions of the Ross-Macdonald model,
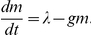
so at equilibrium:
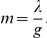
An equivalent expression for the basic reproductive number is then:
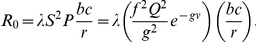
Each set of terms in the models corresponds to a different part of the process that is subject to control: larval ecology and larval control (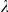), adult blood feeding and survival and adult vector control 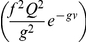, the duration of infection and control by treating infections with drugs (*1/r*), using vaccines or drug chemoprophylaxis to block infection (*b*), and using drugs or vaccines that block transmission from humans (*c*).

Second, not all aspects of a mosquito life cycle affect transmission equally (Table 2). In Macdonald's original formulation of *R_0_*, control effect sizes scaled approximately quadratically (i.e., 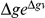) with proportional changes in mosquito survival (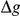); halving mosquito longevity would reduce vectorial capacity by approximately one-fourth [70]. By rewriting *R_0_* (Box 8), it's clear that there are three effects: a mosquito must live long enough to become infected, survive the pathogen latent period, and then survive long enough to give some number of infectious bites. Control effect sizes actually scale with the third power of mosquito longevity (i.e. with 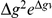), so halving longevity would reduce vectorial capacity to approximately one-eighth of baseline [93]. Increasing mortality and limiting lifespan would also limit the number of eggs laid and, depending on the form of density dependence in aquatic populations, further reduce vectorial capacity. The formula also predicts a quadratic effect of mosquito blood feeding rates and host choice. The effect sizes of mosquito density (larval control), the duration of latency (temperature), the efficiency of transmission (vaccines), and the duration of an infection (drugs) are approximately linear. This is not to say that the effect sizes of control through modes with linear effects are, therefore, small or more difficult to achieve. Indeed, changes in mosquito density over space and time are a leading candidate for the enormous spatio-temporal fluctuations in vectorial capacity, and in some situations, larval control could be highly cost-effective [95]. These effect sizes can be achieved through multiple independent modes of control, and measured directly through changes in vectorial capacity or EIR, or through monitoring infections in host populations. Combined with the variety of metrics for measuring transmission, there is a basis for testing the theory.

**Table 2:** Sensitivity of effect sizes to changes in the underlying parameters is very different.

	% Decrease	*m,b,c,r^−1^*	*a = fQ*	*g*		
				*v* = 10 d	*v* = 15 d	*v* = 20 d
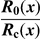	50%	1.5	2.25	3.1	3.7	4.4
	100%	2.0	4.00	7.8	10.9	15.2
	150%	2.5	6.25	17.0	28.0	46.2
	200%	3.0	9.00	34.1	66.5	129.5

Effect sizes are linearly proportional to mosquito density (*m*), infectivity (*b,c*), and the duration of the infectious period (*1/r*), quadratically proportional to human feeding (*a*), and approximately cubically proportional to mosquito survival (*g*) depending on the duration of latency in the mosquito (*v*).

## Discussion

Ross pioneered the early development of a theory for mosquito-borne disease transmission and for the mathematical study of infectious diseases. By describing the parasite life cycle and making simple assumptions about transmission by mosquitoes, Ross was able to make quantitative predictions about the qualitative behavior of malaria epidemics, in particular, the existence of a critical mosquito population density required for transmission. When Ross first wrote down the models, the data did not exist in any form that would allow him to examine patterns. Instead, the models stimulated scientific advances by identifying quantities that were worth measuring and providing a context for interpreting those metrics.

Decades later, following additional contributions by Lotka, Macdonald, Draper, Davidson, Garrett-Jones, Moskovskij, and others, the Ross-Macdonald model had grown into a theory. It was no longer just a mathematical model of transmission—instead, it was a set of deliberately simplified models, concepts, and principles that could help to explain some set of inter-related empirical phenomena linked to mosquito-borne pathogen transmission. The theory included the following: (1) dynamic models of malaria transmission that had been analyzed extensively; (2) formulas for *R_0_* and vectorial capacity; (3) a set of metrics for measuring mosquito-borne pathogen transmission, and well-defined predictions about their quantitative relations; (4) the notion of control effect sizes and sensitivity to specific components of transmission, especially the longevity of adult mosquitoes; (5) predictions about the responses and response timelines of various metrics to control; and (6) extensive application of the theory. The Ross-Macdonald theory of malaria transmission dynamics and control had left many obvious and important questions unanswered, but when the GMEP ended in 1969, it had been applied far more extensively than those of other areas of infectious disease epidemiology. Macdonald's death in 1967, the posthumous publication of his last paper in 1968, and the end of the GMEP marked a major break point for mosquito-borne disease modeling.

Seventy years elapsed between Ross's first trip to Sierra Leone [7] and the first field trial to deliberately measure vectorial capacity and test the Ross-Macdonald theory of dynamics and control [86]. In hindsight, it is possible to identify in the early writings of Ross and Macdonald most of the basic conceptual elements of the theory that eventually emerged. Though they may have had a sense of what their ideas could become, and though they substantially advanced the theory, their work included false starts and erroneous ideas. Ross returned from Sierra Leone without knowing how to measure transmission entomologically and his original reasoning about it was only partially correct [11]. Macdonald's model of superinfection was flawed, and he devoted several pages to the measurement of malaria transmission at equilibrium using concepts that now seem misguided, at best [78]. It would be more accurate to say that Ross and Macdonald were striving for something like the theory that finally emerged, but it took contributions by others to more fully develop the key missing elements.

Although Macdonald had utilized the models to guide the GMEP, his impressive contributions were tainted when the GMEP failed to reach the stated endpoint of global eradication. This failure has been discussed at length; contributing factors included the rigid design of the program with a strong emphasis on implementation that was not matched by adequate investment in research [96]. While the lack of a robust research program made it difficult for the GMEP to respond to the challenges that arose, such as insecticide resistance, the direct cause of the failure of the GMEP was the collapse in funding. Macdonald's formula for *R_0_* and sensitivity analysis on mosquito mortality may have provided an intellectual justification for the DDT-based spraying programs, but it was Fred Soper who was responsible for emphasizing programmatic implementation at the expense of research. This does not fully exonerate Macdonald, because he may have been Soper's accomplice; Macdonald sided with Soper during discussions in 1950 about malaria control in Africa [59].

With the basic elements of a theory in place, mathematical approaches for understanding mosquito-borne pathogen transmission expanded in scope and evolved. Macdonald struggled with the question of immunity to malaria, but he never modeled it himself. A few years after he died, a new malaria model was developed and integrated into the design of a large-scale control trial in Garki, Nigeria [97]. The Garki model corrected Macdonald's flawed notion of superinfection (see Box 6), and it implemented both seasonality and immunity. The Garki model was then validated in Kenya [98], and it has continued to be highly influential in malaria research and prevention. New mathematical models were developed that applied the Ross-Macdonald theory to a range of mosquito-transmitted pathogens, and that explored specific aspects of transmission dynamics in depth. The concept of vectorial capacity was general enough to describe potential transmission of any pathogen by any mosquito, but modeling the dynamics of diseases as different as malaria, dengue, filariasis, and zoonotic arboviruses like West Nile virus presented unique challenges. Questions about measuring transmission, understanding persistence, and establishing response timelines for the control of dengue and other pathogens require accounting for a new set of conceptual issues that did not arise for or from malaria. Differences in dynamics and responses to control could arise because of disparities in vector behavior, ecology and competence, differences in the dynamics of infection, disease, and immunity in vertebrate hosts, or the way the effect sizes of control might scale with the various kinds of heterogeneity that affect transmission. An open question is whether vectorial capacity is the right metric for understanding how to scale vector control or other forms of control across malaria, dengue, and other diseases when the reservoir for infection is very small. It is reasonable to wonder whether mosquitoes or something else may limit potential transmission.

The recent history of mosquito-borne diseases reflects an enormous amount of diversity and creativity, including ideas borrowed from the general theory of mathematical epidemiology. The recent history of modeling mosquito-transmitted pathogens is being summarized separately, as a companion to this paper. Even so, as the development of mosquito-transmitted pathogen models has accelerated in recent years, the dominant influence of the Ross-Macdonald model has become increasingly apparent: most mathematical models of mosquito-transmitted pathogens still utilize many of the assumptions of the Ross-Macdonald model. The strength of the Ross-Macdonald theory is that it is conceptually compelling, despite its simplifying assumptions. The limitations of acquiring information about transmission to apply the model in context and questions about its relevance remain as pertinent as ever. Quantitative tests of the theory continue to suggest that there are large problems yet to be solved. In particular, fluctuations in mosquito populations are extremely difficult to predict over time and space, and important sources of heterogeneity and the spatial and temporal scales of transmission remain poorly characterized. Some of these issues have been explored with models during the last 40 years, but lingering questions make it seem inevitable that when the theory is described at the end of the next century, there will be something new to report.

## Supporting Information

Text S1 Annotated bibliography describing development of the Ross-Macdonald model and some relevant early history of mathematical epidemiology.
(DOC)
