# Supplementary material for: Ross, Macdonald, and a Theory for the Dynamics and Control of Mosquito-Transmitted Pathogens
Source: PLoS Pathog. 2012 Apr 5;8(4):e1002588. doi: 10.1371/journal.ppat.1002588 (PMC3320609; doi:10.1371/journal.ppat.1002588)
Supplement: Text S1 — Annotated bibliography describing development of the Ross-Macdonald model and some relevant early history of mathematical epidemiology. (DOC) [file ppat.1002588.s001.doc]

**History of Mosquito-Borne Disease Modeling, 1897-1969**

Papers have been color-coded as follows:

- Mosquito-borne pathogen modeling papers are in red.
- Advances in measuring transmission are in orange.
- Some historically important mathematical and epidemiological papers are in blue.
- Non-modeling papers of historical interest are in black.

*NOTE:* This annotated bibliography also has a list of references, including some papers published much later that are referenced in the annotation. Many early papers were published several times, and republications of mosquito-borne pathogen modeling papers have been included here and discussed, for completeness. The annotation clarifies the history of publication.

**Annotated Bibliography**

1766 Bernoulli D. Essai d’une nouvelle analyse de la mortalité causée par la petite vérole. Mém Sci Math Phys Acad Roy Paris.

1855 Snow J. *On the mode of communication of cholera*.

1879 **Manson P. On the development of *Filaria sanguinis hominis*, and on the mosquito considered as a nurse.**

1889 En'ko P. On the course of epidemics of some infectious diseases.

1897 **Ross R. On some Peculiar Pigmented Cells Found in Two Mosquitos Fed on Malarial Blood.**

*“On August 16^th^ eight of them [mosquitoes] were fed on a patient whose blood contained fair to few crescents (and also filariae)… The seventh insect was killed on August 20^th^, four days after being fed. On turning to the stomach with an oil immersion, I was struck with the appearance of some cells which seemed to be slightly more substantial than the cells of the mosquito’s stomach usually are…. Each of these bodies contained a few granules of black pigment absolutely identical in appearance with the well-known and characteristic pigment of the parasite malaria… In some cases, they showed rapid oscillations within a small range but did not change their position.*

1899 Ross R. Inaugural Lecture on the Possibility of Extirpating Malaria from Certain Localities by a New Method.

Ross still calls mosquitoes “gnats.”

*“It will be observed that the practicability of eradicating malaria in a locality by the extermination of the dangerous mosquitoes in it depends on a single question – Do these mosquitos breed in spots sufficiently isolated and rare to be dealt with by public measures of repression?”*

*“…the question can be decided only by experiment; and the experiment is well worth making.”*

*“A strong argument to the same effect may be adduced from the general laws of distribution of malaria. The disease is never uniformly distributed even in small areas. Isolated spots, individual plantations or barracks or villages, even single houses, are often known to be more malarious than their surroundings. This argues not only that malaria is not due to the common mosquitos which are found almost every where, but that it is caused by mosquitos which have a distribution similar to that of the disease whose haunts are also comparatively rare and isolated.”*

Ross R. Extermination of Malaria.

*“a single small puddle may supply the dangerous mosquitoes containing several square miles containing a crowded population.”*

[Ross R]*.The Malaria Expedition to Sierra Leone.

*Published from “a correspondent.” Ross later acknowledged authorship.

[Ross R]*. The Malaria Expedition to Sierra Leone: Mosquito-Borne Fever at Wilberforce.

[Ross R]*. The Malaria Expedition to Sierra Leone: Anopheles and its Habits Malarious Foci Localised.

[Ross R]*.The Malaria Expedition to Sierra Leone: Habits of Anopheles Continued.-Possibility of Extirpation.-Explanation of the Old Laws of Malaria.

Ross R. Life history of the parasites of malaria.

1900 Ross R, Annett HE, Austen EE. Report of the Malaria Expedition of the Liverpool School of Tropical Medicine and Medical Parasitology.

The report contains a long discussion of the bionomics of Anopheles mosquitoes (Ross still calls them gnats). A discussion of disease prevention starts on page 37. Ross lists bednets, swatting, repellants, clothing, and the use of fans and screens among other risk factors. On page 40, he discusses attack on the mosquito in its aquatic habitat. In the next section, he discusses the prospects of success in terms of the number of pools and their accessibility.

**Reed W, Carroll J, Agramonte A, Lazear JW. The etiology of yellow fever—a preliminary note.**

1901 MacGregor W, Ross R, Young JM, Fearnside CF. A Discussion On Malaria And Its Prevention.

MacGregor discusses the use of quinine, mosquito netting, and attack on mosquitoes as measures of control.

*“There is probably only one really accurate method by which we can determine the degree of malaria in a given locality, and that is by ascertaining the average time in which a newcomer becomes infected.”*

**Reed W, Carroll J, Agramonte A. The etiology of yellow fever: An additional note. JAMA.**

**Reed W, Carroll J: The Prevention of Yellow Fever.**

1902 Ross R. *Mosquito brigades and how to organize them*.

*“It will be scarcely more easy to gauge the decrease in the number of mosquitoes than to gauge that of malaria.”* He suggests using a mosquito trap.

Ross R. Researches on malaria.

Ross’s Nobel Lecture. Reprinted in 1967 .

1903 Ross R. The thick film process for the detection of organisms in the blood.

Ross explains the “thick film” technique for identifying parasites.

Ross R. An improved method for the microscopical diagnosis of intermittent fever.

1904 Ross R. The anti-malarial experiment at Mian Mir.

Ross’s essay is part 3 of a longer debate about a large experiment that had been conducted in Mian Mir .

Ross presents a cost-effectiveness argument, that malaria was far more expensive than the meager investment “a sum of this magnitude … which for economical reasons may be spent on banishing the disease there.”

Ross’s arguments: “it might not have continued long enough and that the radius of operations might not have been large enough” is a precursor to the model that appeared later that year. The last two paragraphs lay out the case for it:

*“…the broad principles which govern the prophylaxis of malaria… though self-evident enough, require a more or less mathematical treatment for their formal demonstration. The logical basis of the great measure of mosquito reduction is absolute. There is no doubt whatever that in any locality we can reduce mosquitoes to any percentage we please, provided that we arrest their propagation to a sufficient degree within a sufficient radius. This proposition, like the multiplication table, does not require experimental proof and is incapable of disproof.”*

*“Experiment is required, not in support of the general principle, but only in order to obtain certain unknown constants. We still have to determine (a) the radius of operations required to reduce the density of a given species of mosquito to a given percentage: and (b) the percentage of mosquito reduction required in order to obtain ultimately a given percentage of malaria reduction. But experiments directed to this end must be of a true scientific quality; they must be prefaced by a mathematical inquiry and be executed by means of rigid tests applied by the brain as well as by the hand.”*

Ross R: The logical basis of the sanitary policy of mosquito reduction.

Following up on the critique of the experiment at Mian Mir, this manuscript describes the 1^st^ mathematical model of any sort applied to a mosquito-transmitted pathogen. The model describes diffusive movement of adult mosquitoes and the distribution of adult mosquitoes after the removal of larval habitats. There is some flexibility in citing this article because it was also published twice in almost identical form in 1905: in *Science* and the *BMJ*. The 1904 reference comes from the bibliography of Fine.

1905 Brownlee J. Statistical Studies in Immunity. Smallpox and Vaccination.

1906 **Bancroft TL: On the aetiology of dengue fever.**

Hamer W. The evidence of variability and of persistency of type.

Hamer WH. *The Milroy lectures on epidemic disease in England*.

1907 Ross R. The prevention of malaria in British possessions, Egypt, and parts of America.

A nice summary of early larval control efforts. Ross also describes his motives for writing his first mathematical model.

Brownlee J. Statistical Studies in Immunity: The Theory of an Epidemic.

1908 Ross R. *Report on the prevention of Malaria in Mauritius.*

Available as pdf from Google Books. The 1^st^ malaria transmission model. This model was analyzed by Waite , and again by Lotka . For a more recent historical commentary see Fine .

1909 Ross R: *Report on the Prevention of Malaria in Mauritius.* 2^nd^ edn.

Brownlee J. Certain Considerations on the Causation and Course of Epidemics.

Ross R. Malaria in Greece.

1910 Ross R: *The prevention of malaria.*

There was also an American edition. Sections 27-28 (pp 153-164) describe the model (pp. 153-164). In section 31, “The Measurement of Malaria,” Ross comes back to the model. In 31.9 (pp 235-240), he discusses variation in prevalence with respect to age and includes a plot of age-stratified prevalence with the canonical shape. In 31.10 (pp. 240-242), he uses the model to reason through estimation of the inoculation rate. In section 33 (pp 254-257), Ross uses his mathematical model to reason through control. In section 39 (pp. 296-298), Ross argues that malaria can be “eradicated” if the control measures are “reduced to a certain figure; that is, if the new infections can no longer keep pace with the natural recoveries.”

Ross R, Thomson D. Some enumerative studies on malaria fever.

A landmark study relating parasite densities to febrile events, repeated many times since.

Waite H: Mosquitoes and Malaria. A Study of the Relation between the Number of Mosquitoes in a Locality and the Malaria Rate.

*“The ratio of the number of persons affected with malaria to the total population of a district at a given time is called the Malaria Rate of the district at that time. In general, the rate is continually changing owing to (a) new infections, (b) recoveries, (c) emigration and immigration, (d) the birth and death rates, and (e) the extent to which cases are isolated, as well as owing to changes in the mosquito population.*

*As emigration and immigration vary considerably in different localities, and in the same locality at different times, their influence on the malaria rate cannot be satisfactorily dealt with except in particular cases where the necessary statistics are available; neither would results in general terms be of much practical use.”*

Of special note is the postscript, which discusses how his results differ from those of Ross, including the following excerpt: *“The principal points of agreement are: (a) for a given number of anophelines per unit of the population the number of malaria cases will gradually rise or fall to a fixed value at which it will remain stationary,and (b) when the anophelines are less than a certain number (about forty per unit of the population) there can be no stable condition and the malaria cases will gradually decrease and finally disappear.”*

*“The divergence seems to be chiefly due to the difference in the time units employed in the two methods of treatment. Professor Ross has used the month throughout and has taken the value of m constant during each month, while I have used the average time between two consecutive infecting bites as my unit. The fact that m is increased by unity each time a healthy person is bitten by an infected mosquito and is continually being diminished owing to recoveries, fully justifies, in my opinion, the adoption of this unit.”*

1911 Ross R: *The prevention of malaria.* 2^nd^ edn.

London: John Murray. ⇒In the addendum to this edition, Ross presents the 2^nd^ malaria transmission model.

Ross R: Some quantitative studies in epidemiology.

1912 Lotka A. Quantitative studies in epidemiology.

1914 McKendrick AG. Studies on the theory of continuous probabilities, with special reference to its bearing on natural phenomena of a progressive nature.

1915 Brownlee J. On the curve of the epidemic.

Ross R: Some *a priori* Pathometric Equations.

McKendrick AG: The epidemiological significance of repeated infections and relapses.

1916 Ross R: An application to the theory of probabilities to the study of a priori pathometry. Part I.

Brownlee J. On the curve of the epidemic. Supplementary note.

McKendrick A. Applications of the kinetic theory of gases to vital phenomena.

1917 Ross R, Hudson H: An application of the theory of probabilities to the study of a priori pathometry. Part II.

Ross R, Hudson H: An application of the theory of probabilities to the study of a priori pathometry. Part III.

1918 Brownlee J. An investigation into the periodicity of measles epidemics in London from 1703 to the present day by the method of the periodogram.

1919 McKendrick AG. Theory of invasion by infective agents.

1920 Brownlee J. An investigation into the periodicity of measles epidemics in the different districts of London for the years 1890-1912.

McKendrick A. Statistics of Valour and Service.

1921 Ross R: The principle of repeated medication for curing infections.

Martini E: *Berechnungen und Beobachtungen zur Epidemiologie und Bekämpfung der Malaria.*

Lotka wrote a note in Nature about the equations in 1923.

1922 Brownlee J, Young M. The epidemiology of summer diarrhoea.

1923 Lotka A. Contributions to the analysis of malaria epidemiology.

Lotka AJ: Contributions to the analysis of malaria epidemiology. I. General part.

Lotka AJ: Contributions to the analysis of malaria epidemiology. II. General part (continued). Comparison of two formulae given by Sir Ronald Ross.

Lotka AJ: Contributions to the analysis of malaria epidemiology. III. Numerical part.

Sharpe FR, Lotka AJ: Contributions to the analysis of malaria epidemiology. IV. Incubation lag.

Lotka AJ: Contributions to the analysis of malaria epidemiology. V. Summary.

Lotka A: Martini's equations for the epidemiology of immunising diseases.

1926 McKendrick AG: Applications of mathematics to medical problems.

Macdonald G: Malaria in the children of Freetown, Sierra Leone.

1927 Kermack WO, McKendrick AG: A Contribution to the Mathematical Theory of Epidemics.

The three papers by Ross and Hudson are acknowledged on the 7^th^ line and cited in the bibliography.

1928 Ross R: *Studies on malaria.*

1929 Ross R: Constructive Epidemiology.

1931 Ross R, Hudson HP: *A priori pathometry.*

This “book” is a bound copy of the three-part series by Ross and Hudson from 1916-1917 on *a priori* pathometry

1932 Kermack KO, McKendrick AG: Contributions to the mathematical theory of epidemics - ii. The problem of endemicity.

1933 Davey TH, Gordon RM: The estimation of the density of infective anophelines as a method of calculating the relative risk of inoculation with malaria from different species or in different localities.

This is not a modeling paper, but it is one of the first papers to compare metrics of transmission side by side. In section II (pages 29-30), the paper acknowledges Ross and Waite in promoting the quantitative approach to malaria epidemiology. Macdonald analyzes this data in 1950.

Kermack KO, McKendrick AG: Contributions to the mathematical theory of epidemics - iii. Further studies of the problem of endemicity.

1934 Muench H. Derivation of rates from summation data by the catayltic curve.

1936 Martini ECW: *Wege der Seuchen.*

Muench H. The probability distribution of protection test results.

1937 Kermack WO, McKendrick AG. Contributions to the mathematical theory of epidemics: IV. Analysis of experimental epidemics of the virus disease mouse ectromelia.

1939 Earle WC, Pérez M, del Río J, Arzola C. Observations on the course of naturally acquired malaria in Puerto Rico.

Kermack WO, McKendrick AG. Contributions to the mathematical theory of epidemics: V. Analysis of experimental epidemics of mouse-typhoid; a bacterial disease conferring incomplete immunity.

1947 Walton GA: On the control of malaria in Freetown, Sierra Leone. I. *Plasmodium falciparum* and *Anopheles gambiae* in relation to malaria occurring in infants.

This article presents a correct formula for the distribution of the number of concurrent infections (i.e. the multiplicity of infection), the first mathematical consideration of superinfection for malaria.

1950 Macdonald G: The analysis of infection rates in diseases in which superinfection occurs.

This was Macdonald’s first and only time that Macdonald presents a dynamic equation. Several articles have described a discrepancy in the mathematics, and few have adopted Macdonald’s formulation here, but it remains of historical interest.

Macdonald G: The analysis of malaria parasite rates in infants.

This was published back to back with the model on superinfection. They were intended to be published as a pair. Macdonald’s landmark paper that presents an estimate of the duration of the infectious period found by following a cohort of infectious people, and it also presents analysis of several age-stratified cross-sectional prevalence surveys to obtain an estimate of the force of infection, which was compared to the estimated entomological inoculation rate.

1951 Macdonald G: Community aspects of immunity to malaria.

1952 Macdonald G: The analysis of the sporozoite rate.

Macdonald assembled data to examine the dynamics of infection in mosquitoes, including estimates of the druation of the extrinsic incubation period as a function of temperature for both *P. falciparum* and *P. vivax* and estimates of the longevity of mosquitoes.

Macdonald G: The analysis of equilibrium in malaria.

This paper follows up on the previous publication and uses the logic of the previous paper to define the “basic reproduction rate” of malaria for the first time.

1953 Macdonald G: The analysis of malaria epidemics.

Macdonald G, Davidson G: Dose and cycle of insecticide applications in the control of malaria.

Armitage P: A note on the epidemiology of malaria.

Davidson G, Draper CC: Field studies of some of the basic factors concerned in the transmission of malaria.

Draper CC, Davidson G: A new method of estimating the survival-rate of anopheline mosquitoes in nature.

1954 Davidson G. Estimation of the survival-rate of anopheline mosquitoes in nature.

Gillies MT. The recognition of age-groups within populations of Anopheles gambiae by the pre-gravid rate and the sporozoite rate.

1955 Macdonald G. The measurement of malaria transmission.

Macdonald G: A new approach to the epidemiology of malaria.

Davidson G. Further studies of the basic factors concerned in the transmission of malaria.

1956 Macdonald G: Theory of the eradication of malaria.

Macdonald G: Epidemiological basis of malaria control.

Sensitivity of transmission intensity to adult longevity, and the effect size of adult mosquito control with contact pesticides.

1957 W.H.O. Expert Committee on Malaria, Sixth Report.

Macdonald G: *The epidemiology and control of malaria.*

Bailey NTJ: *The mathematical theory of epidemics*.

1959 Macdonald G: The dynamics of resistance to insecticides by Anophelines.

Muench H: *Catalytic models in epidemiology.*

1961 Macdonald G: Epidemiologic models in studies of vectorborne diseases.

1964 Garrett-Jones C: The Human Blood Index of Malaria Vectors in Relation to Epidemiological Assessment.

The term “vectorial capacity” and its formula were first derived here.

Garrett-Jones C: Prognosis for interruption of malaria transmission through assessment of the mosquito's vectorial capacity.

Garrett-Jones C, Grab B: The Assessment of Insecticidal Impact on the Malaria Mosquito's Vectorial Capacity, from Data on the Proportion of Parous Females

Macdonald G, Göeckel GW: The malaria parasite rate and interruption of transmission.

Moshkovsky S. *The dynamics of malaria eradication.*

1965 Macdonald G. Eradication of Malaria.

1967 Moškovskij SD: A further contribution to the theory of malaria eradication.

1968 Macdonald G, Cuellar CB, Foll CV: The dynamics of malaria.

Garrett-Jones C. Epidemiological entomology and its application to malaria.

1969 Garrett-Jones C, Shidrawi GR: Malaria vectorial capacity of a population of Anopheles gambiae: an exercise in epidemiological entomology.

1980 Onori E, Grab B. Indicators for the forecasting of malaria epidemics.

**Some Useful Commentaries & Reviews**

1969 Bruce-Chwatt LJ: Quantitative epidemiology of tropical diseases.

1975 Fine PEM. Ross's *a priori* pathometry - a perspective.

Fine PEM: Superinfection - a problem in formulating a problem

1976 Bruce-Chwatt LJ: Swellengrebel oration: mathematical models in the epidemiology and control of malaria.

1977 Bruce-Chwatt L. Ronald Ross, William Gorgas, and Malaria Eradication.

1978 Service MW. A short history of early medical entomology.

1979 Fine P. John Brownlee and the measurement of infectiousness: an historical study in epidemic theory.

1982 Bailey NTJ: *The biomathematics of malaria.*

Aron JL, May RM. The population dynamics of malaria.

1985 Nedelman J: Some New Thoughts About Some Old Malaria Models - Introductory Review.

Molineaux L: The pros and cons of modelling malaria transmission.

1988 Dietz K: Mathematical models for transmission and control of malaria. In *Principles and Practice of Malaria.*

Dietz K. The first epidemic model: A historical note on PD En'ko.

1991 Koella JC: On the use of mathematical models of malaria transmission.

Anderson RM, May RM. *Mathematical Epidemiology.*

1993 Dietz K: The estimation of the basic reproduction number for infectious diseases.

2000 McKenzie FE: Why model malaria?

Dobson MJ, Malowany M, Snow RW. Malaria control in East Africa: the Kampala Conference and the Pare-Taveta Scheme: a meeting of common and high ground.

2004 McKenzie FE, Samba EM: The role of mathematical modeling in evidence-based malaria control.

Smith DL, McKenzie FE. Statics and dynamics of malaria infection in Anopheles mosquitoes.

2008 Silver JB. *Mosquito Ecology: Field Sampling Methods*. 3^rd^ ed.

**References**

1. Bernoulli D (1766) Essai d’une nouvelle analyse de la mortalité causée par la petite vérole. Mém Sci Math Phys Acad Roy Paris.

2. Snow J (1855) On the mode of communication of cholera. London: John Churchill.

3. Manson P (1879) On the development of *Filaria sanguinis hominis*, and on the mosquito considered as a nurse. J Linn Soc London, ZooI 14: 304-311.

4. En'ko P (1989) On the course of epidemics of some infectious diseases. International journal of epidemiology.

5. Ross R (1897) On some peculiar pigmented cells found in two mosquitos fed on malarial blood. British Medical Journal 2: 1786-1788.

6. Ross R (1899) Inaugural Lecture on the Possibility of Extirpating Malaria from Certain Localities by a New Method. British Medical Journal 2: 1-4.

7. Ross R (1899) Extermination of Malaria: Ind. Med. Gaz. 231-232 p.

8. (1899) The Malaria Expedition to Sierra Leone. British Medical Journal 2: 675-676.

9. (1899) The Malaria Expedition to Sierra Leone: Mosquito-Borne Fever at Wilberforce. British Medical Journal 2: 746.

10. (1899) The Malaria Expedition to Sierra Leone: Anopheles and its Habits Malarious Foci Localised. British Medical Journal 2: 869-871.

11. (1899) The Malaria Expedition to Sierra Leone: Habits of Anopheles Continued.-Possibility of Extirpation.-Explanation of the Old Laws of Malaria. British Medical Journal 2: 1033-1035.

12. Ross R (1899) Life history of the parasites of malaria. Nature.

13. Ross R, Annett HE, Austen EE (1900) Report of the malaria expedition of the Liverpool School of Tropical Medicine and Medical Parasitology. Liverpool: University Press of Liverpool. 1-71 p.

14. Reed W, Carroll J, Agramonte A, Lazear JW (1900) The etiology of yellow fever—a preliminary note. Public Health Pap Rep 26: 37-53.

15. MacGregor W, Ross R, Young JM, Fearnside CF (1901) A Discussion On Malaria And Its Prevention. British Medical Journal 2: 680-690.

16. Reed W, Carroll J, Agramonte A (1901) The etiology of yellow fever: an additional note. JAMA 36: 431-440.

17. Reed W, Carroll J (1901) The Prevention of Yellow Fever. Public Health Pap Rep 27: 113-129.

18. Ross R (1902) Mosquito brigades and how to organize them. London: Longmans, Green.

19. Ross R (1967) Researches on malaria. Nobel Lectures, Physiology or Medicine, 1901-1921. Amsterdam: Elsevier.

20. Ross R (1903) The thick film process for the detection of organisms in the blood. Thompson Yates and Johnston Laboratories Report V: 117-119.

21. Ross R (1903) An improved method for the microscopical diagnosis of intermittent fever. The Lancet 161: 86.

22. Ross R (1904) The anti-malaria experiment at Mian-Mir. British Medical Journal: 1-50.

23. Stephens JWW, James SP, Ross R, Sewell EP, Strachan H, et al. (1904) Discussion on the prophylaxis of malaria. British Medical Journal ii: 629-642.

24. Ross R (1904) The logical basis of the sanitary policy of mosquito reduction. Proceedings of the Congress of Arts and Sciences, St Louis, USA 6: 89.

25. Ross R (1905) The logical basis of the sanitary policy of mosquito reduction. Science 22: 689-699.

26. Ross R (1905) The logical basis of the sanitary policy of mosquito reduction. British Medical Journal i: 1025-1029.

27. Fine PE (1975) Ross's *a priori* pathometry - a perspective. Proc R Soc Med 68: 547-551.

28. Brownlee J (1905) Statistical Studies in Immunity. Smallpox and Vaccination. Biometrika 4: 313-331.

29. Bancroft TL (1906) On the aetiology of dengue fever. Aust Med Gaz 25: 17-18.

30. Hamer W (1906) The evidence of variability and of persistency of type. The Lancet 167: 733-739.

31. Hamer WH (1906) The Milroy lectures on epidemic disease in England. pp. 1-83.

32. Ross R (1907) The prevention of malaria in British possessions, Egypt, and parts of America The Lancet: 879-887.

33. Brownlee J (1907) Statistical Studies in Immunity: The Theory of an Epidemic. Proceedings of the Royal Society of Edinburgh 26: 484-521.

34. Ross R (1908) Report on the Prevention of Malaria in Mauritius. New York: E. P. Dutton &amp; Company. 202 p.

35. Waite H (1910) Mosquitoes and Malaria. A Study of the Relation between the Number of Mosquitoes in a Locality and the Malaria Rate. Biometrika 7: 421--436.

36. Lotka AJ (1923) Contributions to the analysis of malaria epidemiology. II. General part (continued). Comparison of two formulae given by Sir Ronald Ross. Amer J Hyg 3 (Suppl. 1): 38-54.

37. Ross R (1909) Report on the Prevention of Malaria in Mauritius. London: J. & A. Churchill.

38. Brownlee J (1909) Certain Considerations on the Causation and Course of Epidemics. Proceedings of the Royal Society of Medicine 2: 243-258.

39. Ross R (1909) Malaria in Greece. Washington, DC: Government Printing Office. 697-710 p.

40. Ross R (1910) The prevention of malaria. London: John Murray.

41. Ross R, Thomson D (1910) Some enumerative studies on malaria fever. Proceedings of the Royal Society of London Series B, Containing Papers of a Biological Character 83: 159-173.

42. Ross R (1911) The Prevention of Malaria. London: John Murray. 651-686 p.

43. Ross R (1911) Some quantitative studies in epidemiology. Nature 87: 466-467.

44. Lotka A (1912) Quantitative studies in epidemiology. Nature 88: 497-498.

45. McKendrick AG (1914) Studies on the theory of continuous probabilities, with special reference to its bearing on natural phenomena of a progressive nature. Proceedings of the London Mathematical Society 13: 401-416.

46. Brownlee J (1915) On the curve of the epidemic. British Medical Journal.

47. Ross R (1915) Some *a priori* pathometric equations. British Medical Journal i: 546-547.

48. McKendrick AG (1915) The epidemiological significance of repeated infections and relapses. Indian Journal of Medical Research 3: 266-267.

49. Ross R (1916) An application of the theory of probabilities to the study of a priori pathometry. Part I. Proceedings of the Royal Society of London Series a-Mathematical Physical and Engineering Sciences 92: 204-230.

50. Brownlee J (1916) On the curve of the epidemic. Supplementary note. British Medical Journal 1: 799-800.

51. McKendrick A (1916) Applications of the kinetic theory of gases to vital phenomena. Indian J Med Res 3: 667-687.

52. Ross R, Hudson HP (1917) An Application of the Theory of Probabilities to the Study of a priori Pathometry. Part II. Proceedings of the Royal Society of London Series a-Mathematical Physical and Engineering Sciences 93: 212-225.

53. Ross R, Hudson H (1917) An Application of the Theory of Probabilities to the Study of a priori Pathometry. Part III. Proceedings of the Royal Society of London Series a-Mathematical Physical and Engineering Sciences 93: 225-240.

54. Brownlee J (1918) An investigation into the periodicity of measles epidemics in London from 1703 to the present day by the method of the periodogram. Philos Trans R Soc Lond, B, Biol Sci 208: 225-250.

55. McKendrick AG (1919) Theory of invasion by infective agents. Indian Journal of Medical Research 6: 614-632.

56. Brownlee J (1920) An investigation into the periodicity of measles epidemics in the different districts of London for the years 1890-1912. Proceedings of the Royal Society of London Series B.

57. McKendrick A (1920) Statistics of Valour and Service. Nature 104: 660-661.

58. Ross R (1921) The principle of repeated medication for curing infections. British Medical Journal ii: 1-4.

59. Martini E (1921) Berechnungen und Beobachtungen zur Epidemiologie und Bekämpfung der Malaria. Hamburg: Gente.

60. Lotka A (1923) Martini's equations for the epidemiology of immunising diseases. Nature 111: 633-634.

61. Brownlee J, Young M (1922) The epidemiology of summer diarrhoea. Proceedings of the Royal Society of Medicine. pp. 55-74.

62. Lotka A (1923) Contributions to the analysis of malaria epidemiology. Amer J Hyg 3 (Suppl. 1): 1-121.

63. Lotka A (1923) Contributions to the analysis of malaria epidemiology. I. General part. Amer J Hyg 3 (Suppl. 1): 1-36.

64. Lotka AJ (1923) Contributions to the analysis of malaria epidemiology. III. Numerical part. Amer J Hyg 3 (Suppl. 1): 55-95.

65. Sharpe FR, Lotka AJ (1923) Contributions to the analysis of malaria epidemiology. IV. Incubation lag. Amer J Hyg 3 (Suppl. 1): 96-112.

66. Lotka A (1923) Contributions to the analysis of malaria epidemiology. V. Summary Amer J Hyg 3 (Suppl. 1): 113-121.

67. McKendrick AG (1926) Applications of mathematics to medical problems. Proceedings of the Edinburgh Mathematical Society 44: 98-130.

68. Macdonald G (1926) Malaria in the children of Freetown, Sierra Leone. Annals of Tropical Medicine and Parasitology 20: 239-262.

69. Kermack W, McKendrick A (1927) A contribution to the mathematical theory of epidemics. Proceedings of the Royal Society of London Series a-Mathematical Physical and Engineering Sciences 115: 13-23.

70. Ross R (1928) Studies on malaria. London: John Murray.

71. Ross R (1929) Constructive epidemiology. British Medical Journal 1: 673-674.

72. Ross R, Hudson H (1931) A priori pathometry. Harrison and Sons, Ltd, London: 75.

73. Kermack KO, McKendrick AG (1932) Contributions to the mathematical theory of epidemics - ii. The problem of endemicity. Proceedings of the Royal Society of London Series A 138: 55-83.

74. Davey TH, Gordon RM (1933) The estimation of the density of infective anophelines as a method of calculating the relative risk of inoculation with malaria from different species or in different localities. Ann Trop Med Parasit 27: 27-52.

75. Macdonald G (1950) The analysis of malaria parasite rates in infants. Tropical diseases bulletin 47: 915-938.

76. Kermack KO, McKendrick AG (1933) Contributions to the mathematical theory of epidemics - iii. Further studies of the problem of endemicity. . Proceedings of the Royal Society of London Series A 141: 94-122.

77. Muench H (1934) Derivation of rates from summation data by the catayltic curve. Journal of the American Statistical Association 29: 25.

78. Martini ECW (1936) Wege der Seuchen. Stuttgart.

79. Muench H (1936) The probability distribution of protection test results. Journal of the American Statistical Association.

80. Kermack WO, McKendrick AG (1937) Contributions to the mathematical theory of epidemics: IV. Analysis of experimental epidemics of the virus disease mouse ectromelia. J Hyg (Lond) 37: 172-187.

81. Earle WC, Pérez M, del Río J, Arzola C (1939) Observations on the course of naturally acquired malaria in Puerto Rico. The Puerto Rico Journal of Public Health and Tropical Medicine 14: 391-406.

82. Kermack WO, McKendrick AG (1939) Contributions to the mathematical theory of epidemics: V. Analysis of experimental epidemics of mouse-typhoid; a bacterial disease conferring incomplete immunity. J Hyg (Lond) 39: 271-288.

83. Walton GA (1947) On the control of malaria in Freetown, Sierra Leone. I. *Plasmodium falciparum* and *Anopheles gambiae* in relation to malaria occurring in infants. Annals of Tropical Medicine and Parasitology 41: 380-407.

84. Macdonald G (1950) The analysis of infection rates in diseases in which superinfection occurs. Tropical diseases bulletin 47: 907-915.

85. Macdonald G (1951) Community aspects of immunity to malaria. British medical bulletin 8: 33-36.

86. Macdonald G (1952) The analysis of the sporozoite rate. Tropical diseases bulletin 49: 569-586.

87. Macdonald G (1952) The analysis of equilibrium in malaria. Tropical diseases bulletin 49: 813-1129.

88. Macdonald G (1953) The analysis of malaria epidemics. Tropical diseases bulletin 50: 871-889.

89. Macdonald G, Davidson G (1953) Dose and cycle of insecticide applications in the control of malaria. Bulletin of the World Health Organization 9: 785-812.

90. Armitage P (1953) A note on the epidemiology of malaria. Trop Dis Bull 50: 890--892.

91. Davidson G, Draper CC (1953) Field studies of some of the basic factors concerned in the transmission of malaria. Trans R Soc Trop Med Hyg 47: 522-535.

92. Draper CC, Davidson G (1953) A new method of estimating the survival-rate of anopheline mosquitoes in nature. Nature 172: 503.

93. Davidson G (1954) Estimation of the survival-rate of anopheline mosquitoes in nature. Nature 174: 792-793.

94. Gillies MT (1954) The recognition of age-groups within populations of *Anopheles gambiae* by the pre-gravid rate and the sporozoite rate. Ann Trop Med Parasitol 48: 58-74.

95. Macdonald G (1955) The measurement of malaria transmission. Proceedings of the Royal Society of Medicine 48: 295-301.

96. Macdonald G (1955) A new approach to the epidemiology of malaria. Indian J Malariol 9: 261-270.

97. Davidson G (1955) Further studies of the basic factors concerned in the transmission of malaria. Trans R Soc Trop Med Hyg 49: 339-350.

98. Macdonald G (1956) Theory of the eradication of malaria. Bulletin of the World Health Organization 15: 369-387.

99. Macdonald G (1956) Epidemiological basis of malaria control. Bulletin of the World Health Organization 15: 613-626.

100. W.H.O. (1957) Expert Committee on Malaria, Sixth Report. Geneva, Switzerland: World Health Organization.

101. Macdonald G (1957) The epidemiology and control of malaria. London, New York,: Oxford University Press. 201 p. p.

102. Bailey NTJ (1957) The mathematical theory of epidemics. London: Charles Griffin.

103. Macdonald G (1959) The dynamics of resistance to insecticides by anophelines. Rivista di Parassitologia 20: 305-315.

104. Muench H (1959) Catalytic models in epidemiology. Cambridge, Massachusetts: Harvard University Press.

105. Macdonald G (1961) Epidemiologic models in studies of vectorborne diseases. Public Health Rep 76: 753-764.

106. Garrett-Jones C (1964) The Human Blood Index of Malaria Vectors in Relation to Epidemiological Assessment. Bull World Health Organ 30: 241-261.

107. Garrett-Jones C (1964) Prognosis for interruption of malaria transmission through assessment of the mosquito's vectorial capacity. Nature 204: 1173-1175.

108. Garrett-Jones C, Grab B (1964) The Assessment of Insecticidal Impact on the Malaria Mosquito's Vectorial Capacity, from Data on the Proportion of Parous Females. Bull World Health Organ 31: 71-86.

109. Macdonald G, Göeckel G (1964) The malaria parasite rate and interruption of transmission. Bulletin of the World Health Organization 31: 365-377.

110. Moshkovsky SD (1964) The dynamics of malaria eradication Geneva: W. H. O. . 1-17 p.

111. Macdonald G (1965) Eradication of Malaria. Public health reports 80: 870-879.

112. Moškovskij SD (1967) A further contribution to the theory of malaria eradication. Bull World Health Organ 36: 992-996.

113. Macdonald G, Cuellar CB, Foll CV (1968) The dynamics of malaria. Bulletin of the World Health Organization 38: 743-755.

114. Garrett-Jones C (1968) Epidemiological entomology and its application to malaria. Geneva: WHO.

115. Garrett-Jones C, Shidrawi GR (1969) Malaria vectorial capacity of a population of Anopheles gambiae: an exercise in epidemiological entomology. Bull World Health Organ 40: 531-545.

116. Onori E, Grab B (1980) Indicators for the forecasting of malaria epidemics. Bulletin of the World Health Organization 58: 91-98.

117. Bruce-Chwatt LJ (1969) Quantitative epidemiology of tropical diseases. Transactions of the Royal Society of Tropical Medicine and Hygiene 63: 131-143.

118. Fine PEM (1975) Superinfection - a problem in formulating a problem Tropical Diseases Builletin 75: 475-488.

119. Bruce-Chwatt LJ (1976) Swellengrebel oration: mathematical models in the epidemiology and control of malaria. Tropical and geographical medicine 28: 1-8.

120. Bruce-Chwatt L (1977) Ronald Ross, William Gorgas, and Malaria Eradication. The American Journal of Tropical Medicine and Hygiene 26: 1071-1079.

121. Service MW (1978) A short history of early medical entomology. Journal of Medical Entomology 14: 603-626.

122. Fine P (1979) John Brownlee and the measurement of infectiousness: an historical study in epidemic theory. Journal of the Royal Statistical Society Series A.

123. Bailey NTJ (1982) The biomathematics of malaria. Oxford: Oxford University Press.

124. Aron JL, May RM (1982) The population dynamics of malaria. In: Anderson RM, editor. Population Dynamics and Infectious Disease. London, UK: Chapman and Hall. pp. 139-179.

125. Nedelman J (1985) Some New Thoughts About Some Old Malaria Models - Introductory Review. Mathematical biosciences 73: 159-182.

126. Molineaux L (1985) The pros and cons of modelling malaria transmission. Transactions of the Royal Society of Tropical Medicine and Hygiene 79: 743-747.

127. Dietz K (1988) Mathematical models for transmission and control of malaria. In: Wernsdorfer W, McGregor I, editors. Principles and Practice of Malaria. Edinburgh, UK: Churchill Livingstone. pp. 1091-1133.

128. Dietz K (1988) The first epidemic model: A historical note on PD En'ko. Australian Journal of Statistics.

129. Koella JC (1991) On the use of mathematical models of malaria transmission. Acta Trop 49: 1-25.

130. Dietz K (1993) The estimation of the basic reproduction number for infectious diseases. Statistical Methods in Medical Research 2: 23-41.

131. McKenzie FE (2000) Why model malaria? Parasitol Today 16: 511-516.

132. Dobson MJ, Malowany M, Snow RW (2000) Malaria control in East Africa: the Kampala Conference and the Pare-Taveta Scheme: a meeting of common and high ground. Parassitologia 42: 149-166.

133. McKenzie FE, Samba EM (2004) The role of mathematical modeling in evidence-based malaria control. Am J Trop Med Hyg 71: 94-96.

134. Smith DL, McKenzie FE (2004) Statics and dynamics of malaria infection in Anopheles mosquitoes. Malar J 3: e13.

135. Silver JB (2008) Mosquito ecology: field sampling methods. New York: Springer.
